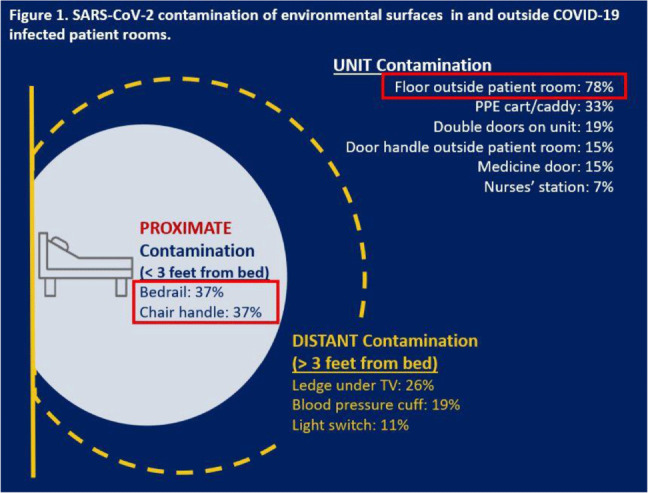# SARS-CoV-2 environmental contamination in COVID-19 patient rooms in a VA medical center

**DOI:** 10.1017/ash.2022.202

**Published:** 2022-05-16

**Authors:** Kristen Gibson, Jennifer Ridenour, Kyle Carver, Julia Mantey, Jane Deng, Lona Mody

## Abstract

**Background:** SARS-CoV-2, the virus causing COVID-19 infection, can significantly contaminate environmental surfaces and can remain viable on surfaces for up to 9 days. Although respiratory route remains the most significant mode of transmission, fomites and environmental sources of infection remain a concern for healthcare personnel who are working in dedicated COVID-19 units. We investigated the extent of detectable SARS-CoV-2 contamination in the environment of COVID-19 patients at a single VA hospital, with the intent of identifying potential high-touch surfaces at risk for viral contamination, which could be used to inform the development of simple COVID-19 prevention strategies. **Methods:** We conducted a cohort study at 1 VA hospital in a unit housing adult veterans admitted with COVID-19 between October and December 2020. In total, 11 swab specimens were collected for PCR analysis (SARS-CoV-2 *env* gene) from environmental surfaces inside and just outside the rooms of COVID-19 patients one time. Retrospective chart reviews were conducted to provide the SARS-CoV-2 epidemiologic context for environmental detection. **Results:** In total, 297 swabs were collected from the unit and environmental areas surrounding 27 hospitalized patients: average age, 72.5 years (range, 34–94); 100% male; 92% non-Hispanic white; average comorbidities, 1.8 (SD, 1.1). Of 297 swabs, 80 (27%) were positive for SARS-CoV-2 and 19 (70%) of 27 patients had at least 1 positive site. The most contaminated site was the floor just outside the patient room (78% positive samples), followed by the patient’s bedrail (37%) and chair handle (37%) (Fig. 1). Traditionally high-touch surfaces, such as the door handle (outside patient room) and the light switch, did not have high positivity rates (<15%). Interestingly, both the personal protective equipment (PPE) cart outside patient’s room (33%) and the double doors leading out of the unit (19%) were positive, which are surfaces often touched with bare hands after handwashing. Analyses of clinical data are underway to examine whether specific care needs, based on activities of daily living disability, comorbidities, and clinical presentation of COVID-19, predict SARS-CoV-2 environmental contamination. **Conclusions:** The presence of environmental contamination by SARS-CoV-2 highlights the importance of transmission via direct or indirect contact. Studies targeting high-risk populations are needed to better understand the transmission of SARS-CoV-2 between infected patients and their environment. Our findings also suggest that handwashing and attention to using disinfecting wipes may mitigate the risk of transmission of virus from surfaces that one might consider safe to touch.

**Funding:** None

**Disclosures:** None

**Figure:**